# A Project to Promote Adherence to Blood Pressure Medication Among People Who Use Community Pharmacies in Rural Montana, 2014–2016

**DOI:** 10.5888/pcd14.160409

**Published:** 2017-06-29

**Authors:** Carrie S. Oser, Crystelle C. Fogle, James A. Bennett

**Affiliations:** 1 Montana Department of Public Health and Human Services, Cardiovascular Health Program, Helena, Montana; 2 Transforming Chronic Care, Bozeman, Montana

## Abstract

**Introduction:**

Pharmacists can assist patients in managing their blood pressure levels. We assessed whether adherence to blood pressure medication improved among people who used community pharmacies in rural Montana after pharmacists initiated consultations and distributed educational materials developed for the Million Hearts Initiative’s “Team Up. Pressure Down.” (TUPD) program.

**Methods:**

From 2014 to 2016, the Cardiovascular Health Program at the Montana Department of Public Health and Human Services conducted a statewide project to evaluate an intervention for adherence to blood pressure medication administered through community pharmacies. After the year 1 pilot, we redesigned the program for year 2 and year 3 and measured the percentage of participating patients who adhered to blood pressure medication. We also conducted a statewide survey to assess pharmacy characteristics, computer-system capabilities, and types of consulting services provided by pharmacists.

**Results:**

Twenty-five community pharmacies completed Montana’s TUPD program: 8 pharmacies in the pilot year, 11 pharmacies in year 2, and 6 pharmacies in year 3. For year 2 and year 3 combined, the percentage of participating patients who achieved blood pressure medication adherence improved preintervention to postintervention from 73% to 89%, and adherence improved in 15 of the 17 pharmacies. The pilot pharmacies identified 3 major barriers to project success: patient buy-in, staff burden in implementing the project, and funding. In the statewide assessment, TUPD-funded pharmacies were significantly more likely than non-TUPD–funded pharmacies to provide prescription synchronization and medication management with feedback to the patient’s physician.

**Conclusion:**

Community pharmacies in rural areas can effectively use brief consultations and standard educational materials to improve adherence to blood pressure medication.

**MEDSCAPE CME**

Medscape, LLC, is pleased to provide online continuing medical education (CME) for this journal article, allowing clinicians the opportunity to earn CME credit.

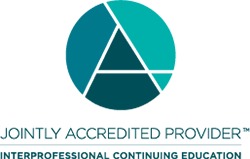
In support of improving patient care, this activity has been planned and implemented by Medscape, LLC, and *Preventing Chronic Disease*. Medscape, LLC, is jointly accredited by the Accreditation Council for Continuing Medical Education (ACCME), the Accreditation Council for Pharmacy Education (ACPE), and the American Nurses Credentialing Center (ANCC), to provide continuing education for the healthcare team.

Medscape, LLC, designates this Journal-based CME activity for a maximum of 1.00 *AMA PRA Category 1 Credit(s)™*. Physicians should claim only the credit commensurate with the extent of their participation in the activity.

All other clinicians completing this activity will be issued a certificate of participation. To participate in this journal CME activity: (1) review the learning objectives and author disclosures; (2) study the education content; (3) take the post-test with a 75% minimum passing score and complete the evaluation at http://www.medscape.org/journal/pcd; (4) view/print certificate.

Release date: June 29, 2017; Expiration date: June 29, 2018

**Learning Objectives**

Upon completion of this activity, participants will be able to:

1. Assess the effectiveness of a pharmacy-based program to improve adherence to antihypertensive drugs in a rural state
2. Distinguish the most common barrier among pharmacies to an adherence program in a rural state
3. Distinguish barriers cited by patients to an adherence program in a rural state
4. Evaluate differences in rural pharmacy practice based on their participation in the antihypertensive adherence program in a rural state

**EDITOR**

Ellen Taratus, MS

Editor, *Preventing Chronic Disease*

Disclosure: Ellen Taratus has disclosed no relevant financial relationships.

**CME AUTHOR**

Charles P. Vega, MD

Health Sciences Clinical Professor, UC Irvine Department of Family Medicine; Associate Dean for Diversity and Inclusion, UC Irvine School of Medicine, Irvine, California

Disclosure: Charles P. Vega, MD, has disclosed the following relevant financial relationships:

Served as an advisor or consultant for: McNeil Consumer Healthcare

Served as a speaker or a member of a speakers bureau for: Shire Pharmaceuticals

**AUTHORS**

Carrie S. Oser, MPH

Cardiovascular Health Program, Montana Department of Public Health and Human Services, Helena, Montana

Disclosure: Carrie S. Oser, MPH, has disclosed no relevant financial relationships.

Crystelle C. Fogle, MBA, MS, RD

Cardiovascular Health Program, Montana Department of Public Health and Human Services, Helena, Montana

Disclosure: Crystelle C. Fogle, MBA, MS, RD, has disclosed no relevant financial relationships.

James A. Bennett, RPh, CDM, CDE

Transforming Chronic Care, Bozeman, Montana

Disclosure: James A. Bennett, RPh, CDM, CDE, has disclosed the following relevant financial relationships:

Served as a speaker or a member of a speakers bureau for: Johnson & Johnson Diabetes Institute

Employed by a commercial interest: Bennett Apothecary Powered by Walgreens in Corinth, Mississippi (part-time diabetes educator/pharmacist)

## Introduction

High blood pressure is a controllable risk factor for cardiovascular diseases (eg, heart disease, stroke) (1). However, patients with hypertension often find it challenging to manage their condition. Barriers to management may include problems with medication adherence, not understanding the seriousness of the condition, or difficulty making lifestyle changes. Community pharmacists can extend the reach of health care providers and assist patients in controlling their hypertension. With ready and consistent access to patients who refill prescriptions monthly, pharmacists are in a position to establish an ongoing relationship with their patients.

A meta-analysis of 7 randomized controlled trials showed that adherence to blood pressure medication increased more in the pharmacist-led interventions than in the control groups (2). In the 6 studies that provided quantitative data, adherence in the intervention groups increased from 56% (203 of 360 participants) to 68% (246 of 360 participants); in the control groups, adherence increased from 59% (190 of 320 participants) to 61% (195 of 320 participants). Another meta-analysis found that 7 of 16 pharmacist interventions significantly increased medication adherence (3); the difference between adherence in the intervention groups compared with the control groups ranged from 8 to 58 percentage points.

Pharmacy-based interventions are also effective in improving medication adherence among people in racial/ethnic minority populations (4,5). We found no studies of pharmacy interventions to improve adherence to blood pressure medication in rural areas. Because of a shortage of primary care providers in rural areas (6), pharmacies in rural areas could play a larger role in improving medication adherence than pharmacies in urban areas. Pharmacists can help identify and overcome barriers (eg, financial difficulties, side effects) that health care providers may not detect during patient visits, which often are infrequent. Pharmacies also can assist patients in managing their blood pressure levels (3,7).

Only 2 interventions that we reviewed (4,8) provided patients with pharmacist consultations and educational materials on blood pressure medication adherence. However, these interventions did not rigorously assess the usefulness of the educational materials.

We evaluated whether patients’ adherence to blood pressure medication improved in rural Montana when we used pharmacy consultations in combination with educational materials that were developed for the Million Hearts Initiative’s “Team Up. Pressure Down.” (TUPD) and were designed for community pharmacists and their patients (9). Our secondary objective was to describe pharmacy characteristics, computer-system capabilities, and types of consulting services provided by pharmacists throughout Montana.

## Methods

This study consisted of 2 components: 1) a 3-year (February 2014–June 2016) intervention to improve adherence to blood pressure medication among people using community pharmacies in rural Montana and 2) a statewide assessment (November 2015–February 2016) of pharmacy characteristics, computer-system capabilities, and types of consulting services provided.

Montana is the fourth largest state geographically but is ranked 48th in the United States for population density, with only 6.8 persons per square mile (10). Much of the state is classified as an area with a shortage of health care professionals or as a medically underserved area (11). According to rural–urban commuting area codes, less than 20% of Montana’s counties had census tracts with a classification of “metropolitan area core” or “metropolitan area high commuting” (12). For the TUPD project, most participating pharmacies were in counties outside these metropolitan areas (13).

### Community pharmacy intervention

In 2014, the Montana Cardiovascular Health (CVH) Program at the Department of Public Health and Human Services (DPHHS) initiated a project with 9 community pharmacies in Montana to conduct and evaluate a blood pressure medication adherence intervention. However, one funded pharmacy did not complete the project because of problems with business structure and staffing. The project used an implementation study design and 3 cohorts (Box 1). The project was supported by the Centers for Disease Control and Prevention (CDC) (14). The Montana DPHHS did not require institutional review board approval because pharmacies submitted only de-identified aggregate data.

**Box 1. Timeline for Team Up. Pressure Down. (TUPD) Blood Pressure Medication Adherence Project in Community Pharmacies, Montana, 2014–2016**

February–June 2014: Pilot project completed with 8 pharmacies

June–August 2014: Formative evaluation

August 2014–June 2015: Year 2 cohort — 11 pharmacies

August 2015–June 2016: Year 3 cohort — 6 pharmacies

November 2015–February 2016: Community pharmacy assessment

The University of Montana’s Skaggs School of Pharmacy provided a list of 258 community pharmacies in Montana. A community pharmacy is designated by the Montana Department of Labor and Industry as a pharmacy that serves customers in a retail setting, such as a pharmacy chain or an independent pharmacy, rather than in an institutional setting, such as a hospital. To recruit pharmacies for the pilot project, the CVH Program mailed an application to all 258 community pharmacies listed, and the Montana Pharmacy Association emailed the announcement to its members. In addition, Montana’s Medicare Quality Innovation Network–Quality Improvement Organization helped recruit pharmacies and disseminate project materials.

Montana DPHHS staff members reviewed 9 applications for the pilot year. The criteria for funding included providing an estimate of the number of patients in the pharmacy who were taking blood pressure medication and the number of patients to be tracked and providing an adequate description of a project plan, including selecting, tracking, and following up with patients. Applicants also were required to describe components that could be continued by the pharmacy without external funding. In the pilot year, all 9 applicants met the application criteria and were funded. Using a similar application and notification process, we funded 2 more cohorts: 11 pharmacies in year 2 and 7 pharmacies in year 3 (one of which did not complete the project because of a staffing shortage). 

#### Pilot project

Each pilot pharmacy was required to recruit at least 25 patients. Participants were required to meet the following minimum criteria: 1) being an adult aged 18 years or older, 2) having had at least one pharmaceutical claim during the previous calendar year (ie, an active pharmacy patient), and 3) having had at least one current prescription for a medication to lower blood pressure. Pharmacies were permitted to customize approaches for identifying and recruiting participants (eg, letters, direct contact).

As part of the project, pharmacies conducted a brief consultation with each participating patient. During the consultation, the pharmacist discussed medication management and changes in lifestyle behavior to help improve the patient’s medication adherence and blood pressure control. We asked the pharmacies to disseminate TUPD’s patient-education materials and information on the Dietary Approaches to Stop Hypertension (DASH) program (15) and to refer smoking patients to the Montana Tobacco Quitline (16). TUPD’s patient-education materials included a blood pressure journal, a medication tracker wallet card, and a medication reminder handout. Additionally, participants received a postcard with information on steps to control blood pressure and a place to list pharmacy and prescription information. TUPD’s pharmacist materials included a pocket discussion guide, a drug-adherence work-up tool (to identify and address patient barriers to taking medication), a blood pressure guide (a quick reference on taking blood pressures manually and interpreting blood pressure readings), and a pharmacy poster.

During the pilot program, pharmacies measured medication adherence by 1) calculating the number of days of refill for a blood pressure medication for each participating patient or 2) using another standard method, such as calculating the percentage of participating patients who achieved blood pressure medication adherence, measured as the proportion of days covered (PDC) by prescription claims as 80% or greater (based on prescription fill date and days of supply). We did not require pharmacies to adhere to a particular method of calculating adherence. Some pharmacies electronically tracked prescription fill dates, and others used an Excel (Microsoft Corp) spreadsheet.

Although the pilot project was designed initially to be implemented during a 10-month period, it was implemented during a 4-month period because of a delay in budget approval. After the conclusion of the pilot program, we obtained feedback from the pilot pharmacies and modified the intervention for year 2 and year 3. We also sought federal guidance on a standard definition of medication adherence (17) and requested additional funding so that we could recruit more pharmacies and increase the funding award to pharmacies as an incentive for them to participate.

#### Year 2 and year 3

In year 2 and year 3, in addition to other program improvements (Box 2), we required pharmacies to use a standardized definition for medication adherence (PDC ≥80%). We received additional funding, which allowed us to double the funding award to pharmacies. We shared lessons learned from the pilot pharmacies with the new pharmacies, emphasized project expectations, and provided additional technical assistance.

**Box 2. Components of Team Up. Pressure Down. (TUPD) in Year 2 and Year 3, Based on Feedback From Pilot Year, Montana, 2014–2016**

• Standardized the definition of medication adherence (17)

• Expanded project time frame from 4 months to 10 months

• Increased the minimum number of patients required to participate from 25 to 35 (year 3 only)

• Doubled the funding award to pharmacies as an incentive for participation

• Offered 2 training options for pharmacists: a home-study blood pressure curriculum and a 1-day hands-on hypertension workshop on accurate blood pressure measurements, current guidelines, lifestyle changes, and medication management

• Provided additional resources to each participating pharmacy: blood pressure cuffs for on-site use and 7-day pill boxes for participating patients

• Provided a sample letter that pharmacists could send to health care providers informing them of their patients’ participation in the project

• Provided a sample press release that pharmacists could send to the local news media to inform their community of the project

• Created an Excel (Microsoft Corp) tracking program for such patient interventions as medication therapy management and lifestyle counseling

• Organized a conference call in which a previously participating pharmacist oriented newly participating pharmacists

• Hired a consulting pharmacist, who owns a community pharmacy, to provide technical assistance and engage pharmacists on a peer-to-peer basis

#### Data collection

The CVH Program collected data from reports filed one month after the project began and final reports. Pre-intervention and postintervention data were collected on medication adherence at the start and end of each of the 3 project periods. The CVH Program developed a final report template that community pharmacies completed at the end of each project period. The final report gave information on barriers, lessons learned, sustainable components, and suggestions for improvement. The final reports also provided data on types of counseling provided and pharmacists’ perceptions of the usefulness of TUPD materials and resources. Lastly, the final report provided aggregate data on the percentage (numerator and denominator) of participating patients who adhered to their blood pressure medication schedule. In addition, for year 2 and year 3, the CVH Program periodically requested interim feedback from the participating pharmacies on progress made and barriers encountered. A consulting pharmacist reviewed the feedback and made suggestions to address barriers as part of his technical assistance.

### Statewide pharmacy assessment

From November 2015 through February 2016, the CVH Program conducted a statewide assessment of community pharmacies to collect data required by CDC to measure grant performance. In October 2015, the CVH Program and a community pharmacist reviewed and revised a survey instrument that the program had designed and used in a statewide assessment in 2013. In November 2015, the Montana Department of Labor and Industry provided a list of licensed community pharmacies. We merged this list and the TUPD recruitment list from the Skaggs School of Pharmacy and eliminated duplicate pharmacies by matching license number, business name, and city, which yielded 259 community pharmacies. The survey, which was mailed, collected information on pharmacy characteristics (the number of pharmacists and pharmacy technicians); computer-system capabilities (acceptance of electronic prescriptions from outside health care facilities, automatic refills on certain maintenance medications, automated refill reminders for blood pressure medication); provision of prescription synchronization (the process of aligning refill dates for all of a patient’s multiple prescriptions); reimbursement of medication therapy management from Mirixa or OutcomesMTM, 2 leading vendors of medication therapy management services in Montana; and the types of consulting services provided by pharmacists. Medication therapy management is a service provided by pharmacists to optimize drug therapy and improve health outcomes.

### Data analysis

For year 2 and year 3 of the intervention, we aggregated the data from the final reports for medication adherence (percentage of participants with PDC ≥80% and total number of participants) from each pharmacy. Details on the calculation of PDC, including definition of terms, unit of analysis, and determination of numerators and denominators, are available elsewhere (17). To generate an overall rate, we aggregated the data on medication adherence by year. The pilot sites were excluded from the medication adherence analysis because they were not required to use a standard definition for medication adherence.

For the community pharmacy assessment, we analyzed data using IBM SPSS Statistics version 21 (IBM Corporation). We used χ^2^ tests to assess any differences in pharmacy and consultation services offered by pharmacists, such as consultation on blood pressure medication adherence, between pharmacies funded by TUPD and pharmacies not funded by TUPD. We also used the nonparametric Mann–Whitney *U* test to compare differences between pharmacies funded by TUPD and pharmacies not funded by TUPD in the number of pharmacists and pharmacy technicians. A *P* value of < .05 was considered significant.

## Results

Twenty-five community pharmacies completed Montana’s TUPD project: 8 in the pilot year, 11 in year 2, and 6 in year 3. All 25 pharmacies submitted a final report. For year 2 and year 3 combined (17 pharmacies), 534 patients completed the TUPD project, with 360 in year 2 and 174 in year 3; the aggregated percentage of participating patients who achieved blood pressure medication adherence increased from 73% pre-intervention to 89% postintervention (Figure). Blood pressure medication adherence improved in 15 of the 17 community pharmacies in year 2 and year 3.

**Figure:**
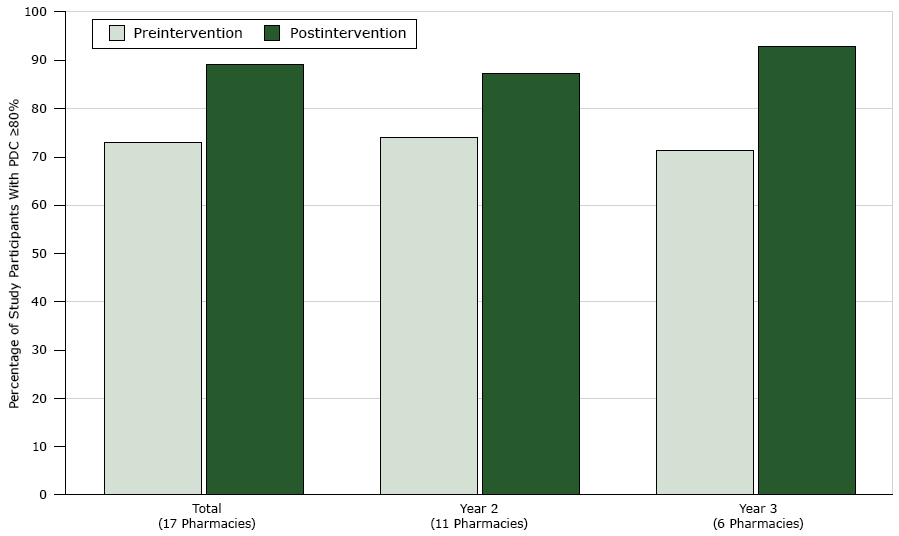
Percentage of patients who achieved blood pressure medication adherence (proportion of days covered [PDC] by prescription claims ≥80%) pre-intervention and postintervention among community pharmacies participating in TUPD (N = 17) in year 2 and year 3, by year and overall, Montana, July 2014–June 2016. Community pharmacies during the pilot year were excluded from this analysis because they were not required to use a standardized definition for medication adherence. Abbreviation: TUPD, Team Up. Pressure Down. **Table:** 

By Year	No. of Participating Pharmacies	Percentage of Study Participants With PDC ≥80%	
		Preintervention	Postintervention
Total	17	73	89
Year 2	11	74	87
Year 3	6	71	93

### Feedback from pharmacists

The pilot pharmacies identified 3 major barriers to project success: patient buy-in, staff burden in implementing the project, and funding. A lack of awareness of the importance of controlling blood pressure, a lack of willingness or interest in project participation, and lack of recognition of the benefits of participation were major obstacles among patients. Staff burden was the most common barrier reported by the pharmacies. Adding another program to a busy schedule was difficult. A lack of time limited the ability of the pharmacists to provide customer service and pharmacy counseling beyond the core task of dispensing medication. In the pilot project, the pharmacists’ suggestions for enhancing the project included developing a template for tracking patients, a notification letter to health care providers, and a checklist of topics to discuss with patients. These resources were added in year 2 and year 3. Other recommendations (a wallet card to log blood pressure values and a survey to obtain patient feedback) will be added in year 4.

Feedback from year 2 and year 3 indicated that involving the entire pharmacy team in the project helped reduce the burden of work on the pharmacists. For example, one pharmacy created a system in which TUPD materials were attached to a patient’s medication refill. When the patient picked up the refill, the pharmacy technician notified the patient that the pharmacist wanted to speak with him or her. Another pharmacy involved the pharmacy technicians in using an alert system (tracking sheet) when a study patient was in the pharmacy so that pharmacists could provide consultations.

TUPD-funded pharmacies reported that TUPD materials and resources were useful. Although all participating pharmacies reported distributing TUPD materials, pharmacists reported only 75% of project participants received these materials because some patients refused them. Pharmacists noted that the wallet card and journal were helpful and of interest to patients, although they noted that some of the materials could be written more concisely. Additionally, 21 pharmacies reported their pharmacists provided lifestyle counseling and medication therapy management to their patients with hypertension.

The pharmacists noted that most of their patients appreciated the extra attention they received during the consultations. Pharmacists adjusted the length of the consultation according to the interest level and needs of each patient. Three pharmacists suggested that the TUPD project may be most suitable for patients with a new diagnosis of hypertension because patients with long-term hypertension had already found ways to manage their condition. One pharmacy lost many project participants because of the participants’ transient employment (oil workers). In addition, 13 pharmacies noted difficulty tracking patients (eg, patient used mail-order or 90-day prescriptions, transferred pharmacies, died, was hospitalized, or moved). Three pharmacies found opportunities to collaborate with patients’ providers to improve blood pressure control.

Twenty pharmacies reported plans to sustain at least one project component to foster medication adherence (eg, measuring blood pressure on-site, offering counseling or medication reviews, providing blood pressure information materials, synchronizing medication, creating a system of automatic refills).

### Statewide pharmacy assessment

The response rate for the community pharmacy assessment conducted was 46% (120 of 259). The average number of pharmacists per pharmacy in Montana was fewer than 3 (Table). We found no significant differences between TUPD-funded pharmacies and non-TUPD–funded pharmacies in the number of pharmacy staff members or pharmacy services related to whether or not automatic refills or refill reminders are provided (Table). TUPD-funded pharmacies were significantly more likely than non-TUPD–funded pharmacies to provide prescription synchronization and medication management with feedback to the patient’s physician. TUPD-funded pharmacies also were more likely than nonfunded pharmacies to report that pharmacists were reimbursed for formal medication therapy management from Mirixa or OutcomesMTM.

**Table:** Characteristics and Services Provided by Community Pharmacies in Rural Areas, by TUPD Funding Status, Montana, 2015–2016^a^

Characteristic or Service	Pharmacies Funded by TUPD (n = 25)	Pharmacies Not Funded by TUPD (n = 95)	*P* Value^b^
**Pharmacy staff, mean (standard deviation)**			
No. of pharmacists	2.6 (1.4)	2.6 (1.4)	.74
No. of pharmacy technicians	3.4 (2.0)	3.1 (2.0)	.64
**Pharmacy services and computer-system capabilities, % (no.)**			
Accept electronic prescriptions from outside health care facilities	100 (25)	95 (90)	.24
Automatically refill selected maintenance medication	88 (22)	79 (74)	.30
Provide automated refill reminders for hypertension medication	60 (15)	50 (47)	.35
Provide prescription synchronization^c^	88 (22)	56 (53)	.003
Obtain reimbursement for pharmacists for formal medication therapy management^d^ from Mirixa or OutcomesMTM^e^	92 (22)	50 (47)	<.001
**Pharmacist counseling, % (no.)**			
Provide consultation services daily	92 (23)	92 (87)	.95
Emphasize importance of following prescribed medication regimen	96 (22)	86 (75)	.21
Assist with medication management and provide feedback to patient’s physician	100 (23)	81 (70)	.02
Provide comprehensive medication review	56 (14)	69 (61)	.21

Abbreviation: TUPD, Team Up. Pressure Down., a program of the Million Hearts initiative (9).

a Data collected through a survey mailed to 259 community pharmacies.

b Calculated by using nonparametric Mann–Whitney *U* test (mean numbers) or χ^2^ test (percentages).

c The process of aligning refill dates for all of a patient’s multiple prescriptions.

d Provided by pharmacist to optimize drug therapy and improve health outcomes.

e Two leading medication therapy management vendors in Montana.

## Discussion

Our findings indicate that it is feasible for community pharmacies in rural areas to provide their patients with brief consultations and TUPD educational materials on how to improve blood pressure medication adherence. Our results are similar to those reported in other studies, which found that pharmacist interventions could significantly improve medication adherence (2–5,8,18,19). The project components in these previous studies were not identical to those in TUPD, however. Some of those interventions provided resources such as a take-home tool kit (4) or blood pressure cuffs for self-monitoring at home (5,7,8) that our project did not provide.

Our study differed from most other studies in that ours focused only on rural pharmacies. Although one study did examine rural Minnesota pharmacies, it was a biennial pharmacy workforce survey of outpatient pharmacies rather than an intervention (20). Also, we did not find any previous study that investigated use of TUPD materials.

Our results suggest that the pharmacies were able to customize the project to fit their needs. In addition, our findings indicate that major components of the project can be integrated into the usual practice of community pharmacies in rural areas. Pharmacies that were already being reimbursed for medication therapy management or that synchronized refills may have been more willing to participate in this project because of their experience in patient consultations.

This project has several limitations. First, our study did not include a control group; however, because this was a project evaluation and not a research project, a control group may not have been needed. Second, pharmacies were not required to collect data on patients’ blood pressure control. We did not institute this requirement because of limited pharmacist time, lack of adequate funding, and difficulty in bringing participants in for measurement. Since we did not require pharmacies to collect data on patients’ blood pressure, we could not conduct additional analyses. However, some participating pharmacies used a blood pressure cuff for on-site measurement, and some made the cuff available to nonparticipating patients. Third, because of the small sample of pharmacies, the results of our study may not be generalizable to all pharmacies. We expect to have a larger sample size for study when additional pharmacies are funded for 2 more years. Fourth, this project assessed only the perceptions of the pharmacists and not those of other stakeholders (pharmacy patients or health care providers). Lastly, because of the annual funding cycle of the CDC grant, we did not investigate long-term medication adherence. Despite these limitations, our project results suggest that community pharmacies in rural areas can use brief consultations and TUPD materials to improve blood pressure medication adherence.

The TUPD project could be expanded to other states that have community pharmacies in rural areas. In year 2, the DPHHS diabetes program broadened the TUPD project by conducting a similar project with 7 of the pilot pharmacies, targeting pharmacy patients taking blood pressure and diabetes medications. Also, in 2016 the state asthma control program recruited 2 of the pilot pharmacies to address asthma medication adherence. This expansion of the TUPD blood pressure approach indicates the willingness of community pharmacies to work on chronic disease management. Future research should evaluate whether the TUPD strategy also improves medication adherence for patients with other chronic conditions such as diabetes or asthma. In addition, research could assess blood pressure control and medication adherence in community pharmacies in rural areas.

## Post-Test Information

To obtain credit, you should first read the journal article. After reading the article, you should be able to answer the following, related, multiple-choice questions. To complete the questions (with a minimum 75% passing score) and earn continuing medical education (CME) credit, please go to http://www.medscape.org/journal/pcd. Credit cannot be obtained for tests completed on paper, although you may use the worksheet below to keep a record of your answers.

You must be a registered user on http://www.medscape.org. If you are not registered on http://www.medscape.org, please click on the “Register” link on the right hand side of the website.

Only one answer is correct for each question. Once you successfully answer all post-test questions, you will be able to view and/or print your certificate. For questions regarding this activity, contact the accredited provider, CME@medscape.net. For technical assistance, contact CME@medscape.net. American Medical Association’s Physician’s Recognition Award (AMA PRA) credits are accepted in the US as evidence of participation in CME activities. For further information on this award, please go to https://www.ama-assn.org. The AMA has determined that physicians not licensed in the US who participate in this CME activity are eligible for *AMA PRA Category 1 Credits™*. Through agreements that the AMA has made with agencies in some countries, AMA PRA credit may be acceptable as evidence of participation in CME activities. If you are not licensed in the US, please complete the questions online, print the AMA PRA CME credit certificate, and present it to your national medical association for review.

## Post-Test Questions

### Study Title: A Project to Promote Adherence to Blood Pressure Medication Among People Who Use Community Pharmacies in Rural Montana, 2014–2016

#### CME Questions

1. You are seeing a high number of patients with hypertension in your rural community, and few health resources are available in your area. You consider teaming up with the local pharmacy to improve adherence to antihypertensive treatment. In the current study by Oser and colleagues, what was the aggregated improvement in treatment adherence in participating centers?
  - 16%
  - 36%
  - 62%
  - 89%
2. You talk to the lead pharmacist about your plan, and she expresses some trepidation. What was the ***most*** commonly cited barrier to pharmacy-based care by pilot pharmacies in the current study?
  - Funding
  - Staff burden
  - Insufficient training
  - Patient buy-in
3. Which of the following were common obstacles for patients receiving care and advice in the current study?
  - Already having achieved perfect or near-perfect adherence
  - Lack of trust in pharmacists' advice
  - Lack of awareness of importance of controlling blood pressure
  - Lack of health insurance coverage for pharmacist visits
4. In the current study, what was the ***biggest*** difference in comparing the practices of pharmacies participating in the Million Hearts Initiative's "Team Up. Pressure Down." (TUPD) program vs pharmacies not participating?
  - Providing counseling services daily
  - Emphasizing the importance of following a prescribed medication regimen
  - Providing a comprehensive medication review
  - Assisting with medication management with feedback to the patient's physician
